# Impact of Depression on Mortality in Patients with Pancreatic Cancer: A Systematic Review

**DOI:** 10.3390/curroncol32090511

**Published:** 2025-09-13

**Authors:** Matthieu Hein, Christelle Bouchart

**Affiliations:** 1 Service de Psychiatrie et Laboratoire du Sommeil, CHU Brugmann, Université Libre de Bruxelles (ULB), 1020 Brussels, Belgium; 2 Laboratoire de Psychologie Médicale et Addictologie (ULB312), Université Libre de Bruxelles (ULB), 1020 Brussels, Belgium; 3 Department of Radiotherapy-Oncology, Institut Jules Bordet—Hopital Universitaire de Bruxelles (H.U.B.), Université libre de Bruxelles (ULB), 1070 Brussels, Belgium; christelle.bouchart@hubruxelles.be; 4 Laboratory of Experimental Gastroenterology, Université Libre de Bruxelles (ULB), Route de Lennik 808, 1070 Brussels, Belgium

**Keywords:** pancreatic cancer, depression, mortality, prevalence, epidemiology

## Abstract

The literature provides evidence of the negative impact of depression on mortality among cancer patients. Depression is also a common comorbidity in pancreatic cancer (PC). The objective of this systematic review was to provide a state-of-the-art overview of the potential role of depression in the excess mortality observed in patients with PC. Based on PRISMA guidelines, a systematic review (PROSPERO: CRD420251135451) was conducted in August 2025 using the Pubmed-Medline and Scopus database. After assessment by two readers of the 325 identified articles, 8 articles (*n* = 143,033) published between 1 January 2010 and 15 August 2025 investigating the specific impact of depression (diagnosed by psychiatric interviews, self-report questionnaires, or diagnostic codes) on mortality in patients with PC (diagnosed by clinical diagnosis or diagnostic codes) were included in this systematic literature review. Articles that were not research studies and were written in a language other than English/French were not included. Risk of bias was assessed using the ROBINS-I tool. A narrative synthesis of the results was performed for the potential impact of depression on mortality in patients with PC. The reported prevalence of depression in this population ranged from 7.4% to 51.8% (seven studies, *n* = 142,983), depending on the studies considered. Most of the included studies (seven studies, *n* = 141,728) consistently reported an increased risk of mortality associated with depression, regardless of cancer stage or treatment received. However, the scientific quality of these studies was generally low, with a significant risk of bias. These results suggest that better integration of depression management in the care of patients with PC could potentially improve clinical outcomes in this high-risk population.

## Simple Summary

In the literature, there are arguments in favor of a particular relationship between depression and pancreatic cancer. Indeed, depression is a frequent comorbidity in patients with pancreatic cancer. Additionally, depressed patients with pancreatic cancer appear to have increased mortality compared to non-depressed patients. However, since the available studies investigating this impact of depression on mortality in patients with pancreatic cancer present important methodological differences, the aim of this systematic review was to provide a state-of-the-art overview of this issue. The prevalence of depression ranged from 7.4% to 51.8% across pancreatic cancer patient samples. Seven of the eight articles selected for this systematic review reported increased mortality associated with comorbid depression in patients with pancreatic cancer, regardless of cancer stage or treatment received. However, the scientific quality of these studies was generally low, with a significant risk of bias. The existence of this potential increased risk of mortality associated with depression suggests that better integration of the management of this psychiatric disorder into the care pathways of patients with pancreatic cancer could potentially improve clinical outcomes in this high-risk population.

## 1. Introduction

Pancreatic cancer (PC)—mainly represented by pancreatic ductal adenocarcinoma—is currently one of the most feared cancers due to its insidious progression, biological aggressiveness and poor prognosis [1,2]. Although it ranks only twelfth in terms of incidence, it is already the fourth leading among causes of cancer death [3,4]. This disproportion between frequency and lethality makes PC a growing public health priority. Projections are alarming: in the United States, PC is expected to become the second leading cause of cancer death by 2030 [5].

The negative consequences of PC are multifaceted, affecting both clinical and psychosocial domains. Symptomatically, this pathology generally manifests itself late with non-specific signs that are frequently overlooked in routine clinical practice—such as abdominal or back pain, weight loss, fatigue, jaundice, and digestive disorders—leading to delayed diagnosis and reduced chances of curative treatment [6,7,8]. Moreover, patients with PC often experience a rapid and severe decline in quality of life, marked by physical deterioration, pain difficult to control, and major impact on mental health [9]. From an economic and societal perspective, results in a substantial loss of potential life years, particularly affecting individuals of working age [10]. In addition, the cost of care associated with this pathology is considerable, due to the complexity of the treatments, the need for multidisciplinary management, and frequent hospitalizations [11,12,13,14]. Additionally, inequalities in access to specialized care may further worsen outcomes in disadvantaged regions or populations [15,16].

From an epidemiological perspective, the age-standardized global incidence of PC increased from 6.3 to 6.6 cases per 100,000 inhabitants between 2010 and 2019 [17,18]. This increase is particularly marked in countries with low or medium socio-demographic index, although industrialized countries are also affected [17,18]. The lifetime risk of developing PC is estimated at 0.89%, with significant regional variations—from 0.15% in Central Africa to over 2% in Western Europe [19,20]. The risk of death is nearly equivalent (0.85%), underscoring the exceptional lethality of this disease [19,20]. Alarmingly, incidence is also increasing among young adults (ages 15–39), with a standardized incidence of 0.2%, a trend likely linked to rising obesity rates and/or environmental pollution such as pesticides [21,22,23,24]. Other identified risk factors include smoking, fasting hyperglycemia, chronic pancreatitis, type 2 diabetes, metabolic syndrome, and genetic predispositions [25].

In terms of prognosis, PC has one of the highest mortality rates among all cancers [26]. Despite decades of research and therapeutic advances, the 5-year survival rate remains below 13% [27]. More than half of patients are diagnosed with metastatic disease, rendering curative approach impossible [28]. Surgery, while potentially curative, is feasible in only about 20% of patients and remains associated with significant morbidity and mortality [29,30,31]. Even among those who undergo surgery for a localized stage, the 5-year survival rate is only 17%, due to the occurrence of locoregional or distant relapses in 86% of cases [32,33]. Furthermore, although systemic treatments—particularly multi-agent chemotherapies—have modestly improved survival, the gains remain limited [34]. For example, in metastatic patients, median survival rarely exceeds 11 months with the most effective protocols and may drop to 2 months outside of clinical trials due to the patient frailty and rapid disease progression [35]. Given these challenges, it is essential to identify additional factors that negatively impact survival in patients with PC, in order to develop new global therapeutic strategies better suited to the complexity of this high-risk population.

In the literature, there is growing evidence of a specific relationship between PC and depression. In fact, depression is more prevalent in PC than in any other gastrointestinal cancer, with reported rates from 33% to 50% [36,37,38]. This elevated prevalence extends beyond formal diagnosis of depression: depressive or anxiety symptoms are reported in up to 76% of patients with PC, compared to only 20% in patients with other types of cancer [36,37,38]. Notably, depressive symptoms often precede the onset of somatic symptoms by several months, suggesting that depression may serve as a prodromal indicator of PC [39]. These findings imply that depression is not merely a psychological reaction to PC diagnosis. Instead, the reverse temporal relationship may point to a bidirectional interaction between these two conditions, potentially mediated by inflammatory, neuroendocrine, or immunological mechanisms [40].

Several studies have demonstrated that depression—whether occurring before or after the diagnosis of PC—is associated with increased all-cause and cancer-specific mortality [41,42,43,44,45,46,47]. Moreover, some evidence suggests that this negative impact of depression on survival persists regardless of tumor stage or treatment received [41,43,44,46,47]. However, despite these converging data, other studies have not found a significant association between depression and mortality in patients with PC [48,49,50]. These discrepancies may be attributed to methodological differences across studies, including variations in the diagnostic criteria for depression (e.g., self-report questionnaires vs. clinical diagnoses), time of assessment (pre- vs. post-cancer diagnosis), duration of follow-up, heterogeneity of the populations studied, and overall data quality [41,42,43,44,45,46,47,48,49,50]. To date, no systematic review of the literature has specifically investigated the potential impact of depression on survival in patients with PC. Given the lack of systematic evaluation of existing literature and the methodological inconsistencies across published studies, the main objective of this systematic literature review was to investigate the potential role of depression in the excess mortality related to PC. The aim was to provide reliable data to support the integration of psychiatric care into the treatment pathway for patients with PC. Additionally, based on the articles selected for assessing the impact of depression on mortality, the secondary objective was to estimate the prevalence of this psychiatric disorder among PC patients, in order to better understand the scope of the issue within this specific population.

## 2. Methods

### 2.1. Article Selection

In compliance with PRISMA (Preferred Reporting Items for Systematic Reviews and Meta-Analyses) 2020 guidelines, a systematic review of the literature was conducted to investigate the specific impact of depression on mortality in patients with PC. The previously unpublished protocol of this review has been registered in PROSPERO (CRD420251135451). The review was carried out between 18 August and 24 August 2025, using the PubMed-Medline and Scopus databases. The search strategy employed the following keyword algorithms:

- (“Pancreatic Neoplasms” [Mesh] or pancreatic cancer) AND (“Depressive Disorder” [Mesh] or depression or mood disorder) AND (“Mortality” [Mesh] or mortality or “Prognosis” [Mesh] or prognosis) for the PubMed-Medline database.
- (TITLE-ABS-KEY (“pancreatic neoplasms” OR “pancreatic cancer”) AND TITLE-ABS-KEY (“depressive disorder” OR “depression” OR “mood disorder”) AND ALL (“mortality“ OR “prognosis”) for the Scopus database.

After excluding duplicate records, this search yielded 325 articles, which were independently assessed by two reviewers. Articles were selected articles based on the following inclusion and exclusion criteria:

- Article investigating the specific impact of depression on mortality in patients with PC.
- Assessment of depression using psychiatric interviews, self-report questionnaires, or diagnostic codes from international classifications systems.
- Diagnosis of PC confirmed through clinical diagnosis or diagnostic codes from international classifications systems.
- Any study design (cross-sectional, longitudinal, prospective, retrospective, interventional, and experimental), except for literature reviews, case reports, opinion papers, animal studies, preprints, and letters to the editor.
- Article published between 1 January 2010 and 15 August 2025.
- Articles written in English or French.
- Articles available in full version.

**Figure 1:**
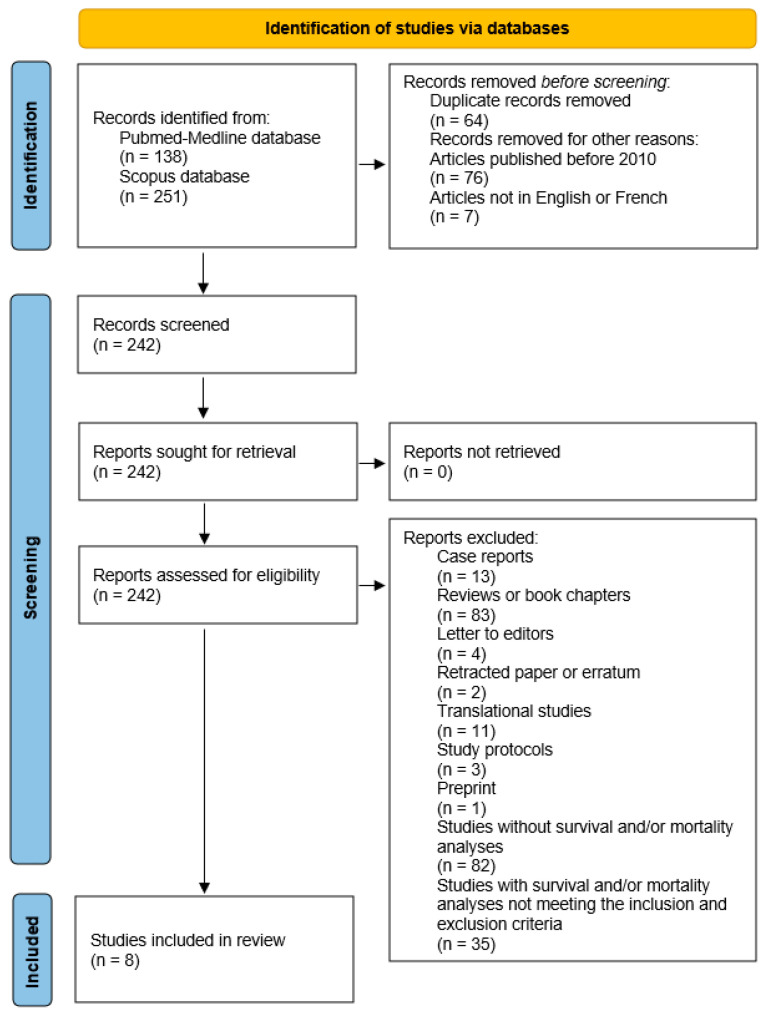
Article selection diagram.

After applying these criteria, eight articles investigating the specific impact of depression on mortality in patients with PC were finally selected from the Pubmed-Medline and Scopus databases for inclusion in this systematic literature review (Figure 1) [41,42,43,44,45,46,47,48]. All discrepancies for article selection were discussed and sorted out by the two reviewers.

### 2.2. Assessment of the Quality and Risk of Bias of the Selected Articles

The quality of the studies selected in this systematic literature review was independently assessed by the two reviewers using the French guidelines issued by the Agence Nationale d’Accréditation et d’Évaluation en Santé (integrated into the Haute Autorité de Santé) [51]. According to these guidelines, three grades of recommendations can be determined based on four levels of scientific evidence (Table 1).

In addition, the risk of bias for each study was independently evaluated by the two reviewers using the ROBINS-I tool (Risk Of Bias In Nonrandomized Studies of Interventions) [52]. This tool assesses bias across seven specific domains: bias due to confounding, bias due to selection of participants, bias in classification of interventions, bias due to deviations from intended interventions, bias due to missing data, bias in measurement of outcomes and bias in selection of the reported results [52].

All discrepancies for quality assessment of articles were discussed and sorted out by the two reviewers.

### 2.3. Data Extraction

After independent work by the two reviewers, data extracted from each of the eight studies selected for the analysis of the available literature were:

(1) Data related to studies: first author name, publication year, country, sample size, recruitment period, study design, grade of recommendation, level of evidence and main limitations.
(2) Data related to patients: age, race, gender, main inclusion/exclusion criteria, stage of PC and detailed treatment of PC.
(3) Data related to exposure: time of depression assessment, depression measurement, prevalence of depression, severity of depression, diagnostic criteria of depression and treatment of depression.
(4) Survival outcome and main confounding factors included in the analyses.
(5) Main results concerning the specific impact of depression on mortality in patients with PC (mean differences for continuous survival data with normal distribution, median differences for continuous survival data with asymmetrical distribution, hazard ratio [HR] with 95% confidence interval for mortality risk associated with depression, odds ratio [OR] with 95% confidence interval for mortality risk associated with depression, and percentage differences for categorical survival data).

Based on the available extracted data, the eight studies selected were grouped for the presentation of the main results into three distinct categories: studies with available data on the prevalence of depression in patients with PC (Table 2 and Table 3), studies with data regarding the impact of depression depending on the time of its diagnosis on mortality in patients with PC (Table 4 and Table 5), and studies with data on the role of worsening depressive symptoms on mortality in patients with PC (Table 6). All discrepancies for data extraction were discussed and sorted out by the two reviewers. All data, codes, and other materials are available in the manuscript and tables.

## 3. Results

### 3.1. Prevalence of Depression in Patients with Pancreatic Cancer

Based on the data extracted from the eight studies included in this systematic literature review, the prevalence of depression among patients with PC ranged from 7.4% to 51.8%, with notable differences depending on the study design—lower prevalence in retrospective cohort or case–control studies (Table 2) and higher in prospective observational studies (Table 3) [41,42,43,44,45,47,48]. In five retrospective cohort or case–control studies that used diagnostic codes (Systematized Nomenclature of Medicine Clinical Terms or International Classification of Diseases) to identify depression [41,42,43,44,48], the reported prevalence ranged from 7.4% to 16.4% (Table 2). However, although the frequency of depression (14.0%) highlighted in the case–control study by Davis et al. (2022) [44] is consistent with the range obtained from the four retrospective cohort studies, it is essential to interpret this result with caution given the specific design of this study. In contrast, the two prospective observational studies that employed the Patient Health Questionnaire-9 (PHQ-9) scale for depression diagnosis, reported significantly higher prevalence rates, ranging from 34.0% to 51.8% [45,47] (Table 3). Additionally, in the prospective observational study by Kitamura et al. (2023) [46], depression was assessed using the Geriatric Depression Scale-Short Form (GDS-SF). However, a diagnostic cut-off was not applied, as the scale was used as a continuous variable. Therefore, the prevalence of depression could not be estimated in that study (Table 3). Finaly, the different clinical characteristics of these studies included in this systematic review are available in Table 2 and Table 3.

### 3.2. Impact of Depression Depending on the Time of Its Diagnosis on Mortality in Patients with Pancreatic Cancer

#### 3.2.1. Impact of Diagnosed Depression Until Confirmation of Pancreatic Cancer Diagnosis

Several studies included in this systematic review found that depression diagnosed prior to or at the time of PC diagnosis was associated with poorer survival [41,43,44,47]. In the study by Boyd et al. (2012) (Table 4) [41], multivariate analyses revealed that patients with locoregional PC and comorbid depression had a significantly higher 2-year mortality compared to non-depressed patients (HR 1.14 [95% CI 1.04–1.26], *p* = 0.006). This negative impact persisted even among those who underwent surgical resection (HR 1.34 [95% CI 1.04–1.73], *p* = 0.023). However, in patients with metastatic PC, depression was not significantly associated with increased 2-year mortality after adjusting for chemotherapy (HR 1.03 [95% CI 0.97–1.09], *p* = 0.324). In the work of Paredes et al. (2021) (Table 4) [43], univariate analyses showed that depression was associated with increased 1-, 3-, and 5-year all-cause and cancer-specific mortality across various subgroups (whole cohort, stage I–II patients, and those undergoing surgery) (*p* < 0.001). Multivariate analyses confirmed that findings, with depressed patients showing higher all-cause (HR 1.10 [95% CI 1.07–1.14], *p* < 0.05) and cancer-specific (HR 1.08 [95% CI 1.04–1.12], *p* < 0.05) mortality. Moreover, in Davis et al. (2022), depression was significantly associated with poorer survival only in patients with metastatic PC (HR 1.32 [95% CI 1.02–1.72], *p* = 0.04), while no significant association was found in those with locoregional disease (HR 1.23 [95% CI 0.82–1.83], *p* = 0.32), even after adjustment for confounding factors in multivariate analyses (Table 4) [44]. Finally, Chen et al. (2025) reported that depression diagnosed concurrently with PC was associated with significantly poorer survival in multivariate analyses (HR 3.61 [95% CI 1.15–11.34], *p* = 0.028) (Table 4) [47].

#### 3.2.2. Impact of Diagnosed Depression During the Period After Pancreatic Cancer Diagnosis

In contrast to the findings of Perry et al. (2022) [48], two other studies—Seoud et al. (2020) and Ji et al. (2023)—reported that depression diagnosed after the onset of PC diagnosis was associated with poorer prognosis [42,45]. In the study by Seoud et al. (2020) (Table 5) [42], multivariate analyses showed that patients with PC and comorbid depression had significantly higher all-cause mortality compared to non-depressed patients (OR 1.18 [95% CI 1.13–1.24], *p* < 0.001). Notably, this study also found that referral to mental health professionals and the implementation of specialized psychiatric care were associated with reduced mortality among depressed patients. In Ji et al. (2023) (Table 5) [45], univariate analyses revealed that depression was significantly associated with higher 1-year mortality in patients with PC (OR 4.39 [95% CI 1.50–12.84], *p* = 0.007). This association remained significant even when analyses were restricted to patients who had undergone surgical resection (OR 5.63 [95% CI 1.12–28.27], *p* = 0.036). Conversely, Perry et al. (2022) did not find a significant association between depression and survival outcomes—including 30-day mortality, 90-day mortality, and overall survival—in their cohort, which consisted exclusively of patients with early-stage PC (stage I or II) who had undergone surgical resection (Table 5) [48].

### 3.3. Impact of Worsening Depressive Symptoms During Pancreatic Cancer Treatment

In their study, Kitamura et al. (2023) found that worsening depressive symptoms during chemotherapy were significantly associated with reduced overall survival in patients with unresectable or recurrent PC (HR 1.35 [95% CI 1.12–1.63], *p* = 0.002) (Table 6) [46].

### 3.4. Quality and Risk of Bias of the Selected Articles

All studies included in this systematic review were rated as having a low level of scientific quality, corresponding to Level 4 evidence and a grade C of recommendation, according to the criteria of the Agence Nationale d’Accréditation et d’Évaluation en Santé (integrated into the Haute Autorité de Santé). Furthermore, the ROBINS-I tool revealed that all these studies exhibited a moderate to severe risk of bias. The most common sources of bias were bias due to confounding, bias due to selection of participants, bias in classification of interventions and bias due to missing data. A detailed assessment of the risk of bias for each study, based on the ROBINS-I tool, is available in Table 7.

## 4. Discussion

In this systematic literature review, we have confirmed that depression is a frequent comorbidity in patients with PC [53]. The prevalence of this mood disorder raged from 7.4% and 51.8%, depending on the studies included [41,42,43,44,45,47,48], consistently confirming that depression is more frequent in PC than in the general population or most other cancers [54,55,56]. We also observed a lower prevalence of depression in retrospective cohort or case–control studies (7.4–16.4%) [41,42,43,44,48], and a higher prevalence in longitudinal observational studies (34.0–51.8%) [45,47]. This discrepancy may be attributed to methodological differences in diagnosing depression. In retrospective cohort or case–control studies, depression was identified using diagnostic codes (Systematized Nomenclature of Medicine Clinical Terms or International Classification of Diseases), whereas in longitudinal observational studies, it was assessed through self-administered questionnaires. The use of diagnostic codes may lead to an underestimation of depression prevalence due to the absence of direct patient verification, increasing the risk of non-reporting or misclassification [57,58]. Conversely, self-report questionnaires may overestimate depression prevalence in longitudinal studies, as these tools only measure the presence of depressive symptoms rather than providing a definitive diagnosis of depression, even when cut-off scores are respected [59,60]. Importantly, none of the studies included in this systematic literature review diagnosed depression through systematic psychiatric interviews, which remain the gold standard for confirming a diagnosis of depression in clinical practice [61,62]. From a pathophysiological and psychopathological perspective, several hypotheses have been proposed to explain the very high prevalence of depression in patients with PC. Biologically, specific pathophysiological mechanisms induced by PC may play a central role due to their demonstrated negative impact on mood regulation. These include (1) inflammatory processes (such as inflammation-mediated tryptophan catabolism via upregulation of the kynurenine pathway and elevated levels of pro-inflammatory cytokines like interleukin-6) that disrupt the hypothalamic–pituitary–adrenal axis and stimulate the secretion of corticotropin-releasing factor [40,63,64,65], (2) hormonal changes (including increased serotonin metabolism) resulting in a depletion of this neurotransmitter in the central nervous system [40], (3) metabolic disturbances (particularly those related to glucose metabolism) inducing altered cerebral glucose metabolism utilization [65], (4) biochemical mechanisms (such as the hyperactivation of β-adrenergic signaling caused by chronic stress associated with PC and production of biogenic amines due to the significant presence of neuropeptides in pancreatic tumors) [40,65], (5) immunological factors (alterations of central serotonergic signaling inducing by a cross-reaction between central serotonergic receptors and antibodies produced in response to PC) [40], and (6) paraneoplastic phenomena (including the production of false neurotransmitters potentially altering brain signaling) [40]. Collectively, these mechanisms contribute to mood disturbances and may underlie the high prevalence of depression observed in patients with PC [40,63,64,65]. In addition to these biological factors, physical and psychosocial consequences of PC—such as pain, gastrointestinal symptoms, physical deterioration, fatigue, emotional distress and social and/or family difficulties—may also contribute to the increased frequency of depression given their direct negative effects on psychological functioning [66,67,68,69,70,71]. Thus, based on these different elements, it is essential to carry out further studies using psychiatric interviews to assess the prevalence of depression in patients with PC. This would provide more reliable data and help determine the need for appropriate screening strategies in this vulnerable population.

Similarly to other cancers [72,73,74], there is evidence of a negative impact of depression on prognosis in PC. With the exception of a single study [48], all studies included in this systematic literature review—whether retrospective cohort, case–control or longitudinal observational—confirmed that depression is associated with higher mortality in patients with PC [41,42,43,44,45,46,47]. Moreover, in studies that included multivariate analyses, this negative impact of depression on mortality generally persisted even after adjusting for treatment received or disease stage [41,43,44,46,47]. To better understand the excess mortality associated with depression in PC, several potential explanations have been proposed in the literature. First, depressed patients with PC are generally diagnosed later with more advanced disease, limiting access to curative treatments and increasing mortality [41,45]. This delay in diagnosis in depressed patients with cancer may result from poorer adherence to medical care and impaired judgment, which hinder timely referral to specialized oncological departments [75,76,77,78]. Second, even after overcoming this obstacle of delayed diagnosis, depression appears to limit access to appropriate therapeutic strategies. Depressed patients with PC receive less chemotherapy, experience more treatment interruptions and undergo surgery less frequently than non-depressed patients, all of which may negatively affect survival [41,43,44,45]. These limitations in treatment access observed for depressed patients are likely due to higher rates of treatment refusal and difficulties in initiating and/or continuing treatments, often driven by reduced quality of life and motivation [79,80,81,82]. Third, depressed patients with PC tend to have a more vulnerable clinical and psychosocial profile, including older age, social isolation, dysfunctional family support, and more frequent comorbidities, all of which being associated with poorer outcomes [41,43,83,84,85,86]. Fourth, depression-related pathophysiological mechanisms—such as inflammation and oxidative/nitrosative stress, decreased immunosurveillance, dysfunctional activation of autonomic nervous system and hypothalamic–pituitary-adrenal axis—may promote cancer progression. These mechanisms can increase tumor invasiveness, reduce immune response, enhance angiogenesis, suppress tumor suppressor gene activity, and inhibit apoptosis [87,88,89]. This biological ling is supported by studies showing advanced stages of PC are more frequently observed in depressed patients [41,45]. Furthermore, these biological factors may help explain the findings of Kitamura et al. (2023), which demonstrated that increased severity of depression during chemotherapy treatment was associated with reduced survival [46]. This suggests that worsening depression may exacerbate the biological processes that drive cancer progression and mortality [90,91]. Interestingly, the only study in this systematic review that did not find an association between depression and increased mortality—Perry et al. (2022)—included only patients with early-stage PC (stage I or II) who underwent curative surgery [48]. The retrospective recruitment of this very particular sub-population of PC likely avoided many of the barriers typically faced by depressed patients [41,42,43,44,45,46,47], such as delayed diagnosis and limited treatment access, probably leading to the neutralization of the excess mortality associated with depression. Given these consistent results, integrating adequate management of depression into care pathways may be crucial for improving clinical outcomes in the high-risk population of patients with PC.

The existence of excess mortality related to depression in patients with PC may open new therapeutic perspectives for this specific population. Indeed, the literature suggests a beneficial effect of both psychotherapeutic and pharmacological treatment for depression on survival in cancer patients. Regarding psychotherapeutic interventions, it has been shown that a reduction in depressive symptoms following group therapy treatment has been associated with longer survival in women with metastatic breast cancer [92]. Moreover, this positive impact of psychotherapy on mortality appears to extend beyond breast cancer, with promising results reported for lymphomas, leukemias, melanomas, gastrointestinal cancers, solid tumors, and non-small cell lung cancers [93]. On the other hand, regarding pharmacological treatments, adequate adherence to antidepressant treatment in depressed cancer patients has been associated with a reduced early mortality [94]. Notably, the effectiveness of pharmacological treatment may depend on the class of antidepressant used, with selective serotonin reuptake inhibitors (SSRIs) showing a greater impact on reducing overall cancer-related morbidity and cancer-specific mortality [95]. Beyond survival outcomes, appropriate mental health treatment—whether psychotherapy or antidepressants—also appears to improve engagement with cancer screening programs among depressed patients [96]. Although data specific to PC are still limited, some studies have demonstrated a reduction in mortality for depressed patients with PC after referral to mental health professionals (36.9% vs. 41.3%, *p* < 0.001) or initiation of antidepressant treatment following mental health contact (37.8% vs. 41.3%, *p* < 0.001) [42]. In addition, preoperative antidepressant treatment in depressed patients with PC has been linked to improved postoperative outcomes, including fewer complications (25.0% vs. 28.0%, *p* < 0.001), fewer extended hospital stays (25.0% vs. 29.0%, *p* < 0.001), and reduced 90-day readmissions (32.0% vs. 36.0%, *p* < 0.001) and mortality (12.0% vs. 15.0%, *p* < 0.001) [97]. On the other hand, it has been highlighted that compared to usual care, the establishment of early palliative care integrating depression control by psychoeducation and/or consultation with a psychiatric specialist was associated with better pain management (reduction in pain scores: 1.5-point vs. 1.0-point, *p* = 0.032) [98], improved quality of life (Functional Assessment of Cancer Therapy-General scale: 81.26 [95% CI: 78.89 to 83.63] vs. 75.90 [95% CI: 73.59 to 78.21], *p* = 0.002) and reduced depressive symptoms (Patient Health Questionnaire-9: 5.55 [95% CI: 4.72 to 6.37] vs. 6.72 [95% CI: 5.91 to 7.53], *p* = 0.048) in patients with PC [99]. Based on these preliminary data and current guidelines for managing depression in oncology patients, more appropriate therapeutic strategies for individuals with PC and comorbid depression should be integrated into their care pathways in close collaboration with mental health professionals to improve their quality of life, their pain management, their adherence to oncological treatments, and their overall prognosis [40]. For patients with PC, psychotherapeutic interventions—such as cognitive behavioral therapy, mindfulness-based therapy, psychoeducation, and supportive-expressive therapies—should be systematically implemented as first-line treatment for mild depression. In case of moderate to severe depression, evidence supports the use of combined approaches, where psychotherapy is paired with pharmacological treatment, rather than relying on either modality alone [100,101,102]. Regarding pharmacological management, selective serotonin reuptake inhibitors (SSRIs) are generally considered the most suitable first-line option in the absence of specific comorbidities, due to their favorable efficacy-to-side-effect ratio and lower risk of interaction with oncological treatments [100,101,102,103]. However, when comorbid conditions such as neuropathic pain are present, serotonin and norepinephrine reuptake inhibitors (SNRIs) may be more appropriate. SNRIs offer comparable efficacy in treating depressive symptoms while providing additional benefits for neuropathic pain complaints [104,105,106]. Nevertheless, despite these potentially beneficial effects of depression treatment on survival, most patients with PC do not currently receive adequate psycho-oncological care that aligns with good clinical practice recommendations [107]. Four main barriers to adequate referral and integration of psycho-oncological care have been identified: (1) the lack of awareness among patients and healthcare providers about the availability of specialized mental health services; (2) the persistent stigma surrounding mental healthcare, even in the context of cancer; (3) the lack of integration of psycho-oncology into routine oncological care pathways and (4) the challenge for health professionals in identifying which patients would benefit most from psycho-oncological support [108,109,110]. Given these barriers, further research is needed to define the most effective therapeutic strategy for managing depression in patients with PC, with the goal of improving survival and overall clinical outcomes.

### Limitations and Future Prospects

This systematic literature review presents several limitations that may affect the interpretation of the results. Methodologically, the review was conducted using only the Pubmed-Medline and Scopus databases, which may have limited the scope of included studies although these two major databases contain the majority of current studies available. Additionally, all selected studies were of low scientific quality and exhibited multiple risks of bias—classified as Grade C, level 4 according to the French recommendations of the Agence Nationale d’Accréditation et d’Évaluation en Santé (integrated into the Haute Autorité de Santé), and as having moderate to severe risk of bias according to the ROBINS-I tool. Beyond the inherent limitations of the review process, the included studies also exhibited significant methodological differences, which may influence the interpretation and generalizability of the results of this systematic review.

The first methodological discrepancy lies in the populations studied. Ji et al. (2023), Kitamura et al. (2023) and Chen et al. (2025) exclusively recruited participants from Asian populations [45,46,47], whereas Boyd et al. (2012), Seoud et al. (2020), Paredes et al. (2021), Davis et al. (2022), and Perry et al. (2022) primarily included North American participants, with a predominance of white individuals [41,42,43,44,48]. This limited ethnic representation may hinder the comparability of results across studies and restrict the applicability of this review’s conclusions to broader or more diverse populations. This is particularly relevant given the well-documented regional disparities in the prevalence of PC and depression, which are influenced by genetic, cultural, and environmental factors [111,112,113,114]. Moreover, all included studies were carried out either in the United States or in Asian countries, where healthcare systems differ substantially. These differences may lead to disparities in access to screening and treatment for both depression and PC, potentially affecting patient outcomes depending on their geographic location [115,116].

The second methodological difference concerns the age of the participants, as some studies focused exclusively on older populations. This emphasis on older patients in the studies by Boyd et al. (2012), Paredes et al. (2021) and Kitamura et al. (2013) [41,43,46] may introduce a significant bias, as their findings may not be generalizable to younger individuals. Compared to younger patients, older adults typically experience higher mortality rates associated with PC and face more complex challenges in the diagnosis and treatment of depression, due to a greater impact on overall functioning and health status [117,118]. The overrepresentation of older participants in these three studies [41,43,46] may therefore limit the comparability of their results with those of other studies that included more age-diverse populations [42,44,45,47,48].

The third methodological difference concerns the substantial disparities in the clinical data reported for PC and/or depression. Notably, none of the included studies provided comprehensive information regarding PC stage and/or treatment, despite these factors having a significant influence on patient prognosis [119,120]. Similarly, data on the severity and/or treatment of depression were either missing or only partially reported in most studies. Furthermore, in all selected studies, depression was diagnosed solely on the basis of diagnostic codes or self-questionnaires, without psychiatric interviews. The absence of these critical clinical elements [95,121] makes it difficult to definitively assess the potential impact of depression on mortality among patients with PC. Finally, there was considerable variation in the timing of depression diagnosis within the care pathway across the included studies. This inconsistency introduces additional heterogeneity, further complicating the comparison and interpretation of their results.

The fourth methodological difference pertains to the variability in outcomes and statistical analyses across the included studies. Specifically, while some studies focused on overall survival [41,44,46,47], others examined all-cause mortality and/or specific mortality outcomes (such as PC-specific mortality, 1-year mortality, 30-day mortality, or 90-day mortality) [42,43,45,48]. These discrepancies in outcome measures complicate direct comparisons between studies and may limit the consistency of the conclusions drawn. Furthermore, although all studies—except that of Ji et al. (2023) [45]—conducted multivariate analyses, several key confounding variables could not be adequately controlled due to missing or incomplete clinical data related to PC and/or depression across all included studies.

In light of these major limitations, it is essential that future research protocols address these methodological gaps to generate higher-quality evidence regarding the potential impact of depression on mortality in patients with PC.

## 5. Conclusions

Depression is a frequent comorbidity in PC, with a prevalence higher than that observed in the general population and in most other cancers. Based on the consistent findings of this systematic literature review, depression appears to be associated with an increased risk of mortality in patients with PC, regardless of cancer stage or treatment received. Moreover, although the data are limited, some promising evidence suggests that adequately treating depression may contribute to a reduction in cancer-related mortality in this population. However, given the existence of significant limitations of the studies included in this systematic review—such as methodological weaknesses, lack of standardized diagnostic approaches, and limited generalizability—it is essential to pursue further scientific research. High-quality prospective studies are needed to confirm the potential role of depression in the excess mortality observed in patients with PC and to guide the development of effective mental health therapeutic strategies.

## Figures and Tables

**Table 1:** Assessment of the quality of studies.

Grade of Recommendations	Level of Scientific Evidence Provided by the Literature
Grade A Established scientific evidence	Level 1 High-powered randomized controlled trials Meta-analyses of randomized controlled trials Decision analyses based on well-conducted studies
Grade B Scientific presumption	Level 2 Low-powered randomized controlled trials Well-conducted non-randomized comparative studies Cohort studies
Grade C Low level of scientific evidence	Level 3 - Case–control studies
	Level 4 - Comparative studies with significant bias - Retrospective studies - Case series - Descriptive epidemiological studies (cross-sectional, longitudinal)

**Table 2:** Clinical characteristics from retrospective cohort or case–control studies.

Studies	Country of Study Study Design Evidence Level Recommendation Level	Population and Recruitment Period	Patient Characteristics	Main Inclusion and Exclusion Criteria	Stage of PC	Treatment of PC	**Depression Assessment**
Boyd et al. (2012) [41]	United States Retrospective cohort study using SEER-Medicare linked data Level 4—Grade C	23,745 patients 1992–2005	78.2 ± 7.0 years 58.8% women 82.8% white	Inclusion criteria: age ≥ 67 years, diagnosis of pancreatic adenocarcinoma, Medicare A/B coverage Exclusion criteria: diagnosis of pancreatic adenocarcinoma by autopsy or death certificate only	31.9% locoregional 68.1% metastatic	Locoregional: - 23.7% surgery (15.0% for depression and 24.4% for no depression) with 51.9% adjuvant chemoradiation (44.4% for depression and 52.2% for no depression) - 41.0% chemoradiation only (32.0% for depression and 41.8%¨for no depression) Metastatic: 27.0% chemotherapy (16.6% for depression and 27.9% for no depression)	Time of assessment: 3 to 27 months before cancer diagnosis Measurement: ICD-9-CM codes for depression Prevalence: 7.9% with pre-existing depression Severity: not assessed Treatment: not assessed
Seoud et al. (2020) [42]	United States Retrospective population-based cohort study using the Explorys database Level 4—Grade C	62,450 patients 1999–2019	Majority > 65 years 67.6% women in pre-depression cancer and 47.9% women in post-depression cancer 79.4% white in pre-depression cancer and 74.2% white in post-depression cancer	Inclusion criteria: diagnosis of PC with or without depression Exclusion criteria: not explicitly stated	Not specified	Not specified	Time of assessment: depression assessed within 6 months, 1 year, and 3 years before and after PC diagnosis Measurement: SNOMED-CT and ICD-10 codes for depressive disorders Prevalence: 16.4% before PC and 13.0% after PC Severity: not assessed Treatment: mental health referral, antidepressants or both
Paredes et al. (2021) [43]	United States Retrospective cohort study using SEER-Medicare linked data Level 4—Grade C	54,234 patients 2004–2016	73 years (IQR: 67–79) 50.2% Female 78.3% White	Inclusion criteria: age ≥ 65 years, diagnosis of pancreatic adenocarcinoma, Medicare A/B coverage Exclusion criteria: diagnosis of pancreatic adenocarcinoma via death certificate/autopsy, unknown diagnosis month, multiple cancers, dementia/personality disorders	Stage I: 7.9% Stage II: 31.0% Stage III: 9.9% Stage IV: 51.2%	Stage I or II: 48.6% surgery (49.5% for no mood disorder and 39.3% for mood disorder) All stages: 36.0% chemotherapy (35.7% for no mood disorder and 40.8% for mood disorder)	Time of assessment: within 3 years before cancer diagnosis Measurement: ICD-9/10 codes for mood disorder Prevalence: 7.4% with pre-existing mood disorder Severity: not assessed Treatment: not detailed
Davis et al. (2022) [44]	United States Retrospective case–control study using electronic medical records Level 4—Grade C	856 patients 2011–2020	71 years (IQR: 63–79) 48.5% Female 74.2% White	Inclusion criteria: diagnosis of PC Exclusion criteria: benign pancreatic lesions or non-PC	Stage I: 8.4% Stage II: 18.9% Stage III: 8.5% Stage IV: 47.8% Incomplete: 16.3%	Chemotherapy (any setting): 72.1% Surgery: 31.1%	Time of assessment: before or after cancer diagnosis Measurement: ICD-9/10 codes, prescription of antidepressants/anxiolytics, clinical documentation of symptoms Prevalence: 14.0% for depression (2.5% before cancer diagnosis) based on ICD-9/10 codes and 20.7% for pre-existing mood disorder Severity: not assessed Treatment: not detailed
Perry et al. (2022) [48]	United States Retrospective cohort study using SEER-Medicare linked data Level 4—Grade C	1305 patients 2009–2013	74.2 ± 5.7 years 56.2% women 88.0% white	Inclusion criteria: stage I–II pancreatic adenocarcinoma, Medicare A/B/D coverage, underwent PC resection Exclusion criteria: incomplete Medicare coverage, non-resectable disease	Stage I: 13.8% Stage II: 86.2%	Surgery: 74.7% pancreaticoduodenectomy (70.7% for mood disorder and 75.5% for no mood disorder), 18.0% distal pancreatectomy (23.1% for mood disorder and 17.1% for no mood disorder), 3.5% total pancreatectomy (0.1% for mood disorder and 3.7% for no mood disorder), 3.9% other (0.1% for mood disorder and 4.0% for no mood disorder) Systemic treatment: 68.1% chemotherapy (62.5% for mood disorder and 69.2% for no mood disorder), 9.7% neoadjuvant (6.3 for mood disorder and 10.4 for no mood disorder), 64.4% adjuvant (60.6% for mood disorder and 65.2% for no mood disorder)	Time of assessment: within 6 months before surgery Measurement: ICD-9 codes for mood disorder and prescription data for mood disorder Prevalence: 16.0% with pre-existing mood disorder Severity: not assessed Treatment: not detailed

PC: pancreatic cancer, SNOMED-CT: Systematized Nomenclature of Medicine Clinical Terms.

**Table 3:** Clinical characteristics from prospective observational studies.

Studies	Country of Study Study Design Evidence Level Recommendation Level	Population and Recruitment Period	Patient Characteristics	Main Inclusion and Exclusion Criteria	Stage of PC	Treatment of PC	Depression Assessment
Ji et al. (2023) [45]	China Prospective longitudinal observational study Level 4—Grade C	114 patients 2021–2022	59.1 ± 9.6 years in patients without depression and 62.0 ± 9.7 years in patients with depression 50.9% women in patients without depression and 35.6% women in patients with depression Chinese population	Inclusion criteria: adults (>18 years) with newly diagnosed pancreatic adenocarcinoma, no prior mental illness, consented to participate Exclusion criteria: prior pancreatic neoplasms, refusal to participate, missing data, prior mental illness, severe complications (Clavien–Dindo IV–V)	T stage ≥ 3: 63.6% in patients without depression and 78.0% in patients with depression Metastasis: 9.1% in patients without depression and 22.0% in patients with depression N0: 67.3% in patients without depression and 69.5% in patients with depression N1–2: 32.7% in patients without depression and 30.5% in patients with depression	Radical surgery with adjuvant chemotherapy: 85.5% in patients without depression and 67.8% in patients with depression Palliative treatments (chemotherapy only): 14.5% in patients without depression and 32.2% in patients with depression	Time of assessment: 2–3 weeks post-discharge (before first chemotherapy) Measurement: PHQ-9 ≥ 5 Prevalence: 51.8% Severity: Grade I (PHQ-9: 5–9), Grade II (PHQ-9: 10–14), Grade III (PHQ-9: 15–19), and Grade IV (PHQ-9: 20–27) Treatment: not specified
Kitamura et al. (2023) [46]	Japan Prospective observational study Level 4—Grade C	50 patients 2015–2020	76 years (range: 70–87) 50.0% women Japanese population	Inclusion criteria: age ≥ 70, histologically confirmed unresectable or recurrent pancreatic adenocarcinoma, performance status 0–2, scheduled for first-line chemotherapy Exclusion criteria: severe dementia, delirium or psychiatric/neurological comorbidities who were deemed ineligible to receive geriatric assessment at the time of the first visit	Unresectable or recurrent	82.0% gemcitabine monotherapy 18.0% gemcitabine + nab-paclitaxel	Time of assessment: before treatment and 2 months after treatment initiation Measurement: Geriatric Depression Scale-Short Form Prevalence: not explicitly stated (depression assessed as a continuous variable) Severity: not assessed Treatment: not specified
Chen et al. (2025) [47]	Taiwan Prospective observational study Level 4—Grade C	279 patients 2021–2023	63.4 ± 11.1 years 45.9% women Taiwanese population	Inclusion criteria: age ≥ 20, diagnosis of PC (stage I–IV), consented to participate Exclusion criteria: Not specified	Stage I: 3.6% Stage II: 15.1% Stage III: 21.5% Stage IV: 55.9% Stage missing: 3.9%	92.5% chemotherapy 27.2% surgery 10% radiation therapy	Time of assessment: at diagnosis and follow-up (months 2, 3, 4, 6, 9, 12) Measurement: PHQ-9 ≥ 10 (clinically depression) Prevalence: 34.0% at baseline Severity: not assessed Treatment: not specified

PC: pancreatic cancer, PHQ-9: Patient Health Questionnaire-9.

**Table 4:** Impact on mortality of diagnosed depression until confirmation of pancreatic cancer diagnosis.

Studies	Survival Outcome	Main Results	Main Limitations
Boyd et al. (2012) [41]	Overall survival Adjusted confounders: age, gender, race, marital status, Charlson comorbidity index, SEER region, surgery, chemotherapy	Patients without depression (3.1 months) had a higher median survival than those with depression (2.1 months) (*p* < 0.001) Locoregional patients without depression (6.6 months) had a higher median survival than those with depression (4.1 months) (*p* < 0.001) Locoregional patients with depression have a higher risk of death within 2 years than those without depression (HR 1.20 [95% CI 1.09–1.32], *p* < 0.001)) even after adjustment for surgery (HR 1.14 [95% CI 1.04–1.26], *p* = 0.006) Resected locoregional patients without depression (15.0 months) had a higher median survival than those with depression (10.6 months) (*p* = 0.003) Resected locoregional patients with depression have a higher risk of death within 2 years than those without depression (HR 1.34 [95% CI 1.04–1.73], *p* = 0.023) Distant patients without depression (2.2 months) had a higher median survival than those with depression (1.7 months) (*p* < 0.001) After adjustment for chemotherapy treatment, depression was no longer a factor associated with higher risk of death within 2 years for distant patients (HR 1.03 [95% CI 0.97–1.09], *p* = 0.324)	No control for all potential confounders, only Medicare patients, possible underreported or misclassified depression following use of ICD-9 codes from claims data, limited staging data for PC, limited follow-up to 2 years for most analyses, exclusion of depression diagnosed within 3 months before cancer diagnosis, retrospective design
Paredes et al. (2021) [43]	All-cause and PC-specific mortality Adjusted confounders: age, sex, race, comorbidity index, marital status, stage, histologic grade, Medicare enrollment cause, SEER registry state	All sample 1-year overall- and cancer-specific survival were higher among individuals without mental illness (31.6%) than among mood disorder patients (23.5%) (*p* < 0.001) 3-year cancer-specific survival was higher among individuals without mental illness (9.5%) than among mood disorder patients (5.7%) (*p* < 0.001) 5-year cancer-specific survival was higher among individuals without mental illness (6.3%) than among mood disorder patients (3.7%) (*p* < 0.001) Stage 1 or stage 2 patients 5-year overall survival was higher among individuals without mental illness (7.4%) than among mood disorder patients (3.8%) (*p* < 0.001) 5-year cancer-specific survival was higher among individuals without mental illness (12.4%) than among mood disorder patients (8.0%) (*p* < 0.001 Surgical resection patients 3-year overall survival was higher among individuals without mental illness (25.8%) than among mood disorder patients (19.4%) (*p* < 0.001) 5-year overall survival was higher among individuals without mental illness (13.3%) than among mood disorder patients (8.5%) (*p* < 0.001) 3-year cancer-specific survival was higher among individuals without mental illness (32.3%) than among mood disorder patients (27.2%) (*p* < 0.001) 5-year cancer-specific survival was higher among individuals without mental illness (20.4%) than among mood disorder patients (14.5%) (*p* < 0.001) Multivariate analyses Mood disorder was associated with significantly higher all-cause mortality (HR 1.10 [95% CI 1.07–1.14], *p* < 0.05) Mood disorder was associated with significantly higher cancer-specific mortality (HR 1.08 [95% CI 1.04–1.12], *p* < 0.05)	No control for all potential confounders, limited to Medicare patients, possible underreported or misclassified mood disorder following use of ICD-9/10 codes from claims data, study focused on mood disorders (not just depression), retrospective design
Davis et al. (2022) [44]	Overall survival Adjusted confounders: age, chemotherapy receipt, pathologic diagnosis, race, marital status, stage at diagnosis, smoking status, income	Patients with stage IV disease and pre-existing mood disorder had worse survival (HR 1.32 [95% CI 1.02–1.72], *p* = 0.04) No significant association was found for localized disease (HR 1.23 [95% CI 0.82–1.83], *p* = 0.32)	No control for all potential confounders, single-center study, limited generalizability, possible underreported or misclassified mood disorder following use of ICD-9/10 codes from claims data, study focused on mood disorders (not just depression), retrospective design
Chen et al. (2025) [47]	Overall survival Adjusted confounders: age, tumor stage, surgery, body mass index, albumin, CRP, neutrophil lymphocyte ratio, platelet lymphocyte ratio	Univariate analyses revealed a significant reduction in overall survival associated with clinically depression (HR 6.25 [95% CI 2.26–17.27], *p* < 0.001) Multivariate analyses confirmed a significant reduction in overall survival associated with clinically depression (HR 3.61 [95% CI 1.15–11.34], *p* = 0.028)	No control for all potential confounders, single-center study, limited generalizability, depression assessment by auto-questionnaire, observational design

PC: pancreatic cancer, ICD: International Classification of Diseases.

**Table 5:** Impact on mortality of diagnosed depression during the period after pancreatic cancer diagnosis.

Studies	Survival Outcome	Main Results	Main Limitations
Seoud et al. (2020) [42]	All-cause mortality Adjusted confounders: age, sex, race	Depression after PC diagnosis was associated with significantly higher all-cause mortality (OR 1.18 [95% CI 1.13–1.24], *p* < 0.001) Patients diagnosed with post- PC depression who were referred to a mental health professional had significantly lower all-cause mortality than patients who were not (36.9% vs. 41.3%) (*p* < 0.001) Treatment of PC patients with depression via a combination of contact with a mental health professional and antidepressant therapy reduced mortality (37.8% vs. 41.3%) (*p* < 0.001) Patients treated with only a mental health referral had similar all-cause mortality rate compared to patients treated with both a mental health referral and antidepressants (36.9% vs. 37.8%) (*p* = 0.591)	Multivariable analyses limited due to database constraints, only patients from the Explorys database, possible underreported or misclassified depression following use of SNOMED-CT and ICD codes, no access to patient-level data, retrospective design
Ji et al. (2023) [45]	1-year mortality Univariate analysis for survival outcomes	In whole sample, patients with depression had higher 1-year mortality rates than patients without depression (30.5% vs. 9.1%) (*p* = 0.007) In resected patients, patients with depression had higher 1-year mortality rates than patients without depression (20.0% vs. 4.3%) (*p* = 0.036) In whole sample, depression was associated with higher risk of 1-year mortality (OR 4.39 [95% CI 1.50–12.84], *p* = 0.007) In resected patients, depression was associated with higher risk of 1-year mortality (OR 5.63 [95% CI 1.12–28.27], *p* = 0.036)	No control for all potential confounders, small sample, low proportion of eligible patients who agreed to participate, single-center study, limited generalizability, self-reported questionnaires for depression assessment, observational design
Perry et al. (2022) [48]	30-day mortality, 90-day mortality, overall survival Adjusted confounders: age, sex, race, Elixhauser comorbidity index, tumor stage, resection type	Patients with pre-existing mood disorder had similar 30-day mortality (3.0% vs. 4.0%) (*p* = 0.035) and 90-day mortality (8.0% vs. 9.0%) (*p* = 0.079) than patients without pre-existing mood disorder Patients with pre-existing mood disorder had similar overall median survival than patients without pre-existing mood disorder (18 months IQR [10–30] vs. 17 months IQR [10–29]) (*p* = 0.990) Patients with pre-existing mood disorder had similar 2-year survival incidence than patients without pre-existing mood disorder (43.0% vs. 39.0%) (*p* = 0.440)	No control for all potential confounders, limited to Medicare patients, possible underreported or misclassified mood disorder following use of ICD-9 codes from claims data, study focused on mood disorders (not just depression), retrospective design

PC: pancreatic cancer.

**Table 6:** Impact on mortality of worsening depressive symptoms during pancreatic cancer treatment.

Studies	Survival Outcome	Main Results	Main Limitations
Kitamura et al. (2023) [46]	Overall survival Adjusted confounders: age, sex, performance status, modified Glasgow prognostic score, tumor site, disease extent, treatment regimen	Increase in Geriatric Depression Scale-Short Form score during chemotherapy was significantly associated with reduced overall survival (HR 1.35 [95% CI 1.12–1.63], *p* = 0.002)	No control for all potential confounders, small sample, single-center study, limited generalizability, reliance on self-reported data for depression assessment, observational design

**Table 7:** Evaluation of biases according to the ROBINS-I tool.

Studies	D1	D2	D3	D4	D5	D6	D7	Global Risk
Boyd et al. (2012) [41]	Moderate	Moderate	Moderate	Low	Moderate	Low	Low	Moderate
Seoud et al. (2020) [42]	Severe	Moderate	Moderate	Low	Moderate	Moderate	Moderate	Severe
Paredes et al. (2021) [43]	Moderate	Moderate	Moderate	Low	Moderate	Low	Low	Moderate
Davis et al. (2022) [44]	Moderate	Moderate	Moderate	Low	Moderate	Low	Moderate	Moderate
Ji et al. (2023) [45]	Severe	Moderate	Moderate	Low	Moderate	Moderate	Low	Severe
Kitamura et al. (2023) [46]	Moderate	Moderate	Low	Low	Moderate	Low	Low	Moderate
Chen et al. (2025) [47]	Severe	Moderate	Moderate	Low	Moderate	Low	Low	Severe
Perry et al. (2022) [48]	Moderate	Moderate	Moderate	Low	Moderate	Low	Low	Moderate

D1: bias due to confounding; D2: bias due to selection of participants; D3: bias in classification of interventions; D4: bias due to deviations from intended interventions; D5: bias due to missing data; D6: bias in measurement of outcomes; D7: bias in selection of the reported results.
